# Utility of Polymerase Chain Reaction Versus Serology-Based Testing in Immunocompromised Patients With *Bartonella henselae* Infection

**DOI:** 10.1093/ofid/ofad130

**Published:** 2023-03-18

**Authors:** Ankit Kumar, Mohammed Abdulqader, Darrell A McBride

**Affiliations:** Internal Medicine, Geisinger Medical Center, Danville, Pennsylvania, USA; Internal Medicine, Geisinger Medical Center, Danville, Pennsylvania, USA; Infectious Diseases, Geisinger Medical Center, Danville, Pennsylvania, USA

**Keywords:** *Bartonella*, immunocompromised, infection, PCR, serology

## Abstract

*Bartonella henselae* serologies can be nondiagnostic in immunocompromised individuals due to impaired humoral immunity. Blood polymerase chain reaction (PCR) is more useful diagnostically in immunosuppressed persons. We discuss 3 cases: 2 solid organ transplant (SOT) recipients and 1 person with human immunodeficiency virus (HIV) with positive blood PCR despite negative serology.

*Bartonella henselae* is transmitted to a person via the scratch of a cat contaminated with flea feces or by the lick of an infected cat over the person’s open wound [1]. Severe manifestations of *B henselae* infection are reported in immunocompromised persons with human immunodeficiency virus (HIV) and in solid organ transplant (SOT) recipients [2, 3]. Given the fastidious nature and limitations in testing, diagnosis requires a high index of suspicion. In immunocompromised persons, serology can be misleading and result negative despite active infection. Decreased sensitivity of *Bartonella* indirect immunofluorescent assay (IFA) (75%) is reported in people with HIV compared to 82%–95% in an immunocompetent group [4]. This may be attributed to impaired humoral response or delayed rise in antibodies. Therefore, we argue that polymerase chain reaction (PCR) testing (tissue or blood) should be preferred or used in addition to serology in immunocompromised persons. Here, we describe 3 cases of *B henselae*–mediated disease in immunocompromised individuals: 2 SOT recipients and 1 person with HIV diagnosed with positive PCR and negative serology.

## CASE 1

A 57-year-old male cat owner with a history of renal transplant in 2018 presented with fever (39.4°C), abdominal pain, and diarrhea. On presentation, his vitals were temperature of 39.2°C and blood pressure (BP) of 80/60 mm Hg. His physical examination was pertinent for diffuse abdominal tenderness and no lymphadenopathy or skin lesions. He was treated with cefepime. Mycophenolate mofetil (MMF) was held, but tacrolimus and prednisone were continued. Computed tomography (CT) showed new multiple indeterminate hypodense lesions in the liver. His laboratory examinations were pertinent for cell count of 3.31 K/µL, hemoglobin of 11.8 g/dL, creatinine of 1.8 mg/dL, and carbon dioxide of 18 mmol/L. His urinalysis, chest radiograph, methicillin-resistant *Staphylococcus aureus* (MRSA) PCR nasal swab, gastrointestinal pathogen panel, *Clostridium difficile* toxin assay, blood cryptococcal antigen, blood and urine cultures, acid-fast bacilli (AFB) blood culture, blood fungal cultures, Epstein-Barr virus (EBV) plasma PCR, cytomegalovirus (CMV) plasma PCR, and Fungitell (1,3)-β-D-glucan assay all resulted negative. Due to persistent fever, he was broadened to vancomycin and piperacillin-tazobactam. He had a Doppler ultrasound of the allograft kidney, which showed well-perfused kidneys. On day 2, multiplanar multisequential magnetic resonance imaging of the liver with contrast showed innumerable T2 bright lesions in the spleen and liver.

On day 3, he had a temperature of 40°C, heart rate (HR) of 123 bpm, BP of 157/85 mm Hg, and respiratory rate (RR) of 32 breaths per minute. Repeat blood culture, respiratory panel, and chest radiograph were negative. He was initiated on micafungin and remained on piperacillin-tazobactam and vancomycin. Nasal MRSA PCR was negative and vancomycin was discontinued. *Bartonella* blood PCR and IFA serologies were obtained. An ultrasound-guided core needle biopsy was collected and sent for cytology, staining, and cultures for bacteria, fungi, and AFB. The cytology showed several foci of inflammatory infiltration in the lobules, histiocytes with some lymphocytes and neutrophils, similar to granulomas with no significant portal inflammation and fibrosis. Immunostaining for CMV and special stains for Grocott's Methenamine Silver (GMS) and AFB were negative for microorganisms.

On day 5, *Bartonella* PCR resulted positive for *B henselae*. Bartonella serology was negative and the patient was switched to oral doxycycline and rifampin. His fever curve improved. On day 9, he was discharged on oral rifampin for 2 weeks and doxycycline for 3–6 months.

## CASE 2

A 57-year-old female cat owner with history of remote heart transplant for advanced heart failure on chronic immunosuppression (tacrolimus, prednisone, MMF, and periodic plasmapheresis) presented to the emergency department with vomiting, abdominal pain, and diarrhea worsening over 2 weeks. On presentation, BP was approximately 65/42 mm Hg and HR was in the 120s. She had no reported fevers. She was fluid resuscitated with improvement in BP. Her CT imaging of the abdomen showed multiple hypodense lesions in the spleen concerning for infectious etiology. Infection workup with blood cultures, urinalysis, *B henselae* and *Bartonella quintana* (PCR, immunoglobulin M [IgM]/immunoglobulin G [IgG]), Q fever IgG/IgM with reflex titer, urine histoplasma antigen, and serum CMV PCR were sent. She was started on cefepime, vancomycin, and micafungin. Her tacrolimus and prednisone were continued and MMF was held. On day 4, the cultures, *Bartonella* serologies, and Q fever serologies resulted negative, and cefepime and vancomycin were discontinued.

On day 6, *Bartonella henselae* serum PCR resulted positive; *B quintana* was not detected. Micafungin was discontinued and doxycycline 100 mg was initiated for 90 days. The patient was diagnosed with peliosis hepatis due to *B henselae*. She was subsequently transferred to Presbyterian Hospital in New York City as her primary transplant care was centered there.

## CASE 3

A 29-year-old man with a history of HIV on antiretroviral therapy (bictegravir/emtricitabine/tenofovir alafenamide) with last absolute CD4 count 155 cells/µL, undetectable viral load, Kaposi sarcoma (biopsy confirmed in January 2021), and *Pneumocystis jirovecii* pneumonia (PJP) presented to the emergency department for evaluation of fever. He had no known cat exposure.

On admission, vitals were temperature 38.7°C, BP 125/79 mm Hg, HR 117 bpm, and RR 23 breaths per minute. His infectious workup revealed leukocytosis (11.05 K/µL), procalcitonin of 0.29 g/mL, and lactate of 1.0 mmol/L; the respiratory panel was positive for adenovirus, negative blood cultures, and urine culture. Cefepime and vancomycin were started empirically.

On day 1, he continued to have intermittent fevers (maximum temperature 39.3°C). He was continued on antiretroviral therapy, vancomycin, cefepime, and trimethoprim-sulfamethoxazole for PJP prophylaxis. His repeat absolute CD4 count was 119 cells/µL with HIV RNA load of 37 copies/mL. His CT of chest, abdomen, and pelvis revealed innumerable ill-defined nodules within lungs with perilymphatic distribution that were noted in the chest CT a month prior, as well as enlarged retroperitoneal, inguinal, periportal, and portocaval lymph nodes. His bronchoscopy cultures from prior admission resulted with *Mycobacterium avium* complex; he was started on ethambutol and azithromycin. Vancomycin and cefepime were discontinued. Given the persistent intermittent fevers (temperature of 40°C), leukocytosis (peak 27.9 K/µL), and lymphadenopathy, there was a concern for possible lymphoma, *Bartonella*, and EBV infection. IFA *Bartonella* species serologies (IgG, IgM) and EBV blood PCR were sent and resulted negative. On day 6, blood PCR for *Bartonella* was sent.

He underwent left inguinal lymph node core needle biopsy and the pathology report showed histiocytes predominantly with rare lymphocytes with extensive necrosis background.

On day 7, the patient was discharged and postdischarge, the blood *Bartonella* PCR resulted positive for *B henselae*. Two weeks later, the patient was admitted for acute hypoxic respiratory failure requiring intubation. He was treated with doxycycline for the *B henselae* infection. He progressed to respiratory failure and shock requiring increasing vasopressor support. He was transitioned to comfort care and subsequently died on day 9. Autopsy showed death due to respiratory failure with Kaposi sarcoma as the primary cause.

## DISCUSSION

Given the vague symptoms and limitations of testing, diagnosis of *B henselae* requires a high clinical suspicion. Available diagnostic testing includes culture, histopathology, PCR, and serology. Given the low yield of fastidious *B henselae* in culture, serological testing is often clinically utilized. Serology with IgG titer >1:256 is strongly suggestive of recent or active infection [2]. These titers can be falsely low in immunosuppressed individuals and thus misleading. In cases 2 and 3, there was negative IFA serology despite active infection. Serology was not obtained in case 1. For both cases 2 and 3, serologies remained negative 2 months from initial diagnosis. Our serology and PCR testing for *Bartonella* disease were standard commercial tests universally used at our institution.

Twenty-eight SOT recipients with definitive *B henselae* infection were identified in 2 literature reviews [5, 6]. Similar to our 2 cases, the immunosuppressive regimen of these patients included prednisone (73%), MMF (60%), tacrolimus (47%), sirolimus (40%), and Muromonab (OKT) (3%). Each immunosuppressive medication has the potential to cause humoral dysfunction. Chronic glucocorticoids increase immunoglobulin catabolism and inhibit interleukin 4 and anti-CD40 mediated B-lymphocyte gene somatic hypermutation [7–9]. MMF profoundly inhibits antibody response in renal transplant, which is well noted with vaccinations [10]. Calcineurin inhibitors have direct humoral suppression by inhibiting naive B-cell proliferation and plasmablast differentiation and indirect suppression of T-helper cells [11].

HIV individuals had diminished antibody titers due to aberrant hyperproliferation of immature/transitional B cells due to increased serum levels of interleukin 7 [12]. Through these mechanisms, there may be an inadequate serologic titer rise or a delayed titer response in immunosuppressed persons, resulting in false-negative IFA serologic testing.

Bergmans at el noted PCR testing positive in 86.4% in patients with cat-scratch disease [12]. Similarly, in 17 out of 30 SOT recipients who received PCR testing, 15 of these 17 (88%) tested positive for *Bartonella* infection [5, 6]. Of the 15 with positive PCR, 87% (7/8) had positive tissue PCR, 71% (5/7) had positive lymph node PCR, and 100% (4/4) had positive blood/serum PCR [5, 6]. Three of 7 seronegative patients had positive tissue PCR [5, 6]. Another study found that 7.9% (5/63) of seronegative patients were positive for blood PCR, showing increased sensitivity from 21.3% using IFA alone to 27.5% with combined IFA and real-time blood PCR [13]. Therefore, PCR testing is preferred in SOT recipients and can increase the yield of diagnosis.

In conclusion, immunocompromised persons with suspected *Bartonella*-mediated infection should undergo PCR testing as initial serologies can be misleading.

## Notes

***Patient consent.*** This study does not include factors necessitating patient consent.
